# Human milk oligosaccharides combine with *Bifidobacterium longum* to form the “golden shield” of the infant intestine: metabolic strategies, health effects, and mechanisms of action

**DOI:** 10.1080/19490976.2024.2430418

**Published:** 2024-11-21

**Authors:** Shuo Yang, Junwu Cai, Qian Su, Qiaohui Li, Xiangchen Meng

**Affiliations:** Key Laboratory of Dairy Science, Ministry of Education, Northeast Agricultural University, Harbin, China

**Keywords:** Human milk oligosaccharides, *Bifidobacterium longum*, metabolic strategies, health effects, mechanisms of action

## Abstract

Human milk oligosaccharides (HMOs) are the third most important nutrient in human milk and are the gold standard for infant nutrition. Due to the lack of an enzyme system capable of utilizing HMOs in the infant intestine, HMOs cannot be directly utilized. Instead, they function as natural prebiotics, participating in the establishment of the intestinal microbiota as a “bifidus factor.” A crucial colonizer of the early intestine is *Bifidobacterium longum* (*B. longum*), particularly its subspecies *B. longum* subsp. *infantis*, which is the most active consumer of HMOs. However, due to the structural diversity of HMOs and the specificity of *B. longum* strains, studies on their synergy are limited. An in-depth investigation into the mechanisms of HMO utilization by *B. longum* is essential for applying both as synbiotics to promote early intestinal development in infants. This review describes the colonization advantages of *B. longum* in the infant intestinal tract and its metabolic strategies for HMOs. It also summarizes recent studies on the effect and mechanism of *B. longum* and HMOs in infant intestinal development directly or indirectly through the action of metabolites. In conclusion, further structural analysis of HMOs and a deeper understanding of the interactions between *B. longum* and HMOs, as well as clinical trials, are necessary to lay the foundation for future practical applications as synbiotics.

## 1. Introduction

After birth, infants face the daunting task of transitioning from a sheltered environment in the womb to the outside world filled with various microbes. Breastfeeding provides strong support for infants to better adapt to this process. Breast milk is thought to have evolved to provide the best nutrition for newborns, being rich in carbohydrates, proteins, fats, minerals, immune factors, and other bioactive substances. Breastfeeding benefits the host’s immune system by providing nutrients to the microbes, leading to a healthier immune–microbial relationship.^1^

Human milk oligosaccharides (HMOs) are the third most abundant nutrient in breast milk after lactose (55–70 g/L) and lipids (16–39 g/L). HMOs act as prebiotics, bridging the gap between the host and microorganisms, and their content changes dynamically with lactation, such as a total of 17.7 g/L of HMOs in colostrum, 13.3 g/L in transitional milk, and 11.3 g/L in mature milk.^2^ HMOs are a unique class of non-digestible carbohydrates present in breast milk that are highly resistant to gastric acid and digestive enzymes, allowing them to reach the colon directly. The degradation of HMOs is closely correlated with the fecal microbial composition, epithelial barrier function, and intestinal immune function of the infant’s intestine.^3,4^ Due to the diversity of glycosidic bonds, more than 200 HMOs have been discovered, with more than 160 structures elucidated.^5,6^ With the continuous progress of modern separation analysis techniques, more HMO structures are waiting to be analyzed.

The intestinal tract of breastfed infants is usually marked by a high concentration of *Bifidobacterium*, a gram-positive anaerobic bacterium commonly distributed in the intestinal tract of animals, which can compete as potential probiotics to inhibit the growth of pathogenic bacteria.^7^ Studies have shown that *Bifidobacterium* deficiency is associated with the initiation of inflammation and immune dysregulation in the infant organism.^8^ Therefore, *Bifidobacterium* is also considered the “keystone” for balancing the intestinal microbiota in breastfed infants.^9^ *Bifidobacterium longum* (*B. longum*) is one of the dominant bacteria in the infant gut, which is divided into three subspecies: *B. longum* subsp. *infantis*, *B. longum* subsp. *longum and B. longum* subsp. *suis*. *B. longum* subsp. *infantis* and *B. longum* subsp. *longum* are mainly related to infant health, showing HMOs degrading ability.^10^ In our previous studies, the fecal microbiota of breastfed infants from different regions of China was analyzed, and the results showed significant geographical differences in the structure of the intestinal microbiota of breastfed infants, with significant aggregation in the same region. Although there were significant differences in the content of HMOs in different regions, *B. longum* was the dominant species in infant feces, which may indicate an association between *B. longum* and HMOs.^11^

This review focuses on the dominate subspecies of *B. longum* (*B. longum* subsp. *infantis* and *B. longum* subsp. *longum*) in the infant’s gut, their metabolic strategies for utilizing HMOs, and how the interaction with HMOs contributes to the regulation of intestinal health. This review aims to enhance the understanding of the mechanisms of interaction between HMOs and *B. longum* in the intestine and provide a referenceable synbiotic to develop specific infant foods.

## 2. How does *B. longum* become dominant in the intestines of infants?

### 2.1. *Transmission of* B. longum

**Figure 1.**
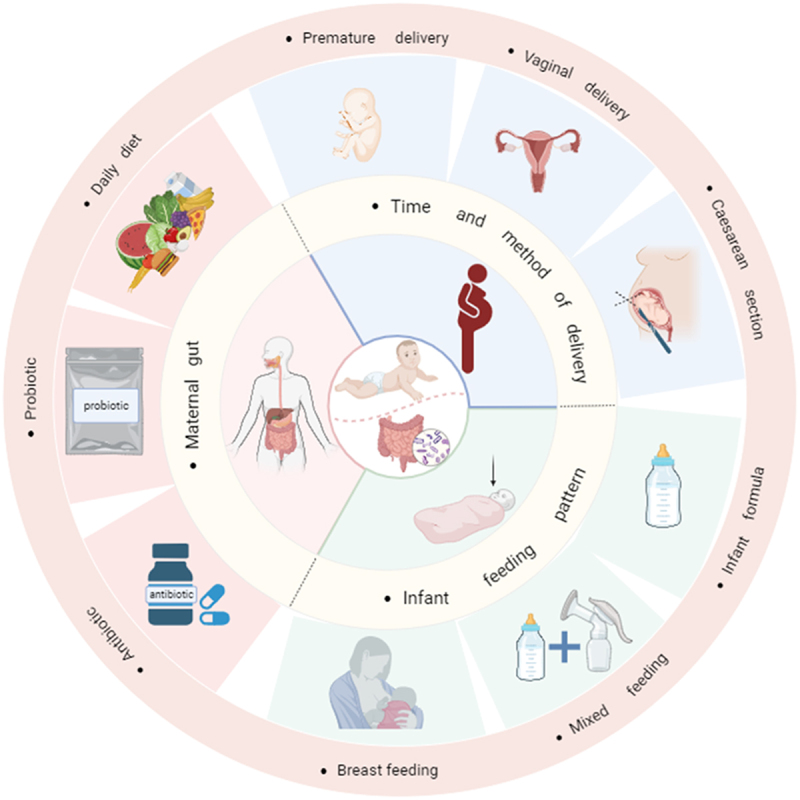
Factors influencing the composition of the gut microbiota in infants. Daily diet, probiotics or antibiotics enter the digestive system through the mother’s mouth and affect the infant in the womb. At birth, preterm birth, vaginal birth or cesarean section affect the infant’s initial microbiome to varying degrees. After birth, infant formula feeding or mixed breastfeeding, exclusive breastfeeding transfers maternal microbiome through the gut-mammary pathway, and microbiome from external contact together make up the infant’s gut microbiome. Image was drawn by Biorender.

An infant’s gut microbiota is initially established primarily during birth and feeding, a process known as vertical transmission. Vertically transmitted strains are thought to be more persistent and stable in the infant’s intestine. *B. longum* is believed to be one of the species that colonizes via this route. It has been shown that the placenta can transfer HMOs from the maternal to the fetal circulation, providing a basis for *B. longum* colonization.^12^ Maternal microbiota undergoes adaptive changes during pregnancy, with progesterone increasing the abundance of *Bifidobacterium*. Factors such as maternal gut microbiome, timing and mode of delivery, and infant feeding patterns influence microbial transmission to varying degrees in the construction of the infant’s gut microbiota (Figure 1).^13,14^

The mode of delivery significantly affects the gut microbiota composition of infants, and our lab previously analyzed the intestinal microbiota of 60 infants born by different delivery methods and found a higher abundance of *Bifidobacterium* in the intestines of vaginally delivered infants compared to those delivered by cesarean section.^15^ Shao et al. analyzed the intestinal microbiota of full-term infants born by different delivery methods and their mothers and found that the bacterial strain transmission rate between vaginally delivered infants and their mothers was as high as 74.39% compared to the cesarean section (C-section) group (12.56%), and the *Bifidobacterium* in the intestinal tract of vaginally delivered infants has been stable at a high level since birth. Besides, *B. longum* was transmitted more frequently from mother to child.^16^ Similarly, Makino et al. found that transmission of *B. longum* appears to be infrequent and delayed in infants born by C-section, as well as in premature infants.^17,18^

Ferretti et al. sampled different body sites of 25 mother-infant pairs and showed that *Bifidobacterium* in the mother’s gut can be stably transferred to the infant’s gut. Compared with other sites, colonization by gut-transmitting strains was more persistent, suggesting that intestinal transmission is a more important mode of vertical transmission.^19^ Consistent with this result, Makino et al. also discriminated 270 strains isolated from eight mother-infant pairs and found that 11 of these strains of *B. longum* subsp. *longum* were monophyletic in mother-infant feces. This further confirmed that these strains were transferred from the intestine of the mother to that of the infant.^20^

Recent studies have shown that there is a gut-mammary pathway in the mother, and secretory immunoglobulin A (SIgA) coated *B. longum* playing a key role in this pathway. These bacteria can be transported from the mother’s gut to the mammary gland and then transferred to the infant’s gut through breastfeeding.^21^ The traditional viewpoint is that breast milk is a sterile biological body fluid, but recent studies have shown that breast milk is rich in microorganisms, with 25% to 30% of an infant’s gut microbiota derived from breast milk. Although they constitute only a small fraction of the colonizing bacteria, they are the pioneer bacteria in the infant’s gut.^22^ However, the enteromammary pathway that promotes the acquisition of *B. longum* by infants is controversial. Researchers have shown that exclusive breastfeeding, as well as later stages of breastfeeding, are associated with a higher abundance of bifidobacteria in breast milk. This could be the result of retrograde flow of bifidobacteria from the infant through the oral cavity, thereby necessitating further investigation into the role of breast milk in unidirectional vertical transmission of bifidobacteria.^23^

In addition, *B. longum* has also been increasingly shown to be horizontally transferable. Studies suggest that bifidobacteria can be transmitted between breastfed infants. However, it is worth noting that most of these studies have focused on very preterm infants. The relatively weakened state of very preterm infants may make them more susceptible to acquiring horizontally transferable strains.^24,25^ In our opinion, the role of horizontal transfer in the acquisition of *B. longum* in healthy infants needs further investigated.

### 2.2. *Extracellular secretions of* B. longum

*B. longum* is an exceptional colonizer of the human intestine. Research on *B. longum* subsp. *longum* strains in the intestine of infants showed that strains confirmed to colonize and persist as early as 90 days after birth were still present at six of age.^26^ Beyond maternal transmission, *B. longum* secretes various extracellular substances that enhance colonization.

One such substance is adhesin, a series of surface proteins that promote attachment to intestinal cells or the extracellular matrix. Iguchi et al. analyzed *B. longum* subsp. *longum* and identified a variant protein that may bind specifically to various carbohydrate structures on the human intestinal wall, facilitating host-specific manner.^27^ Xiong et al. employed PSORTdb to identify FimM as a surface adhesin located to the surface of *B. longum* BBMN68, and then sequence similarity analysis using BLASTP showed that homologous of FimM was found in 23 strains of *B. longum*. It is suggested that FimM is a common surface adhesin in *B. longum* .^28^ While numerous studies have examined adhesin production by *B. longum* in vitro, further research is needed to confirm its effects in vivo.

Extracellular vesicles (EVs), nanovesicles with a phospholipid bilayer secreted by the cell membranes of living cell organisms, also play a role in the colonization of intestinal bacteria. Nishiyama et al. discovered that *B. longum* NCC2705 released numerous EVs into the extracellular environment, primarily composed of cytoplasmic proteins, including mucin-binding proteins. Recombinant expression of these proteins in *Escherichia coli* and fixation on microbeads demonstrated the ability to adhere to the intestinal tract of mice, indicating that EVs secreted by *B. longum* NCC2705 promoted its colonization.^29^ Additionally, EVs contain various polysaccharide-degrading enzymes that may facilitate colonization by promoting carbohydrate decomposition. However, further studies are needed to explore this aspect comprehensively.^30^

Exopolysaccharide (EPS) secreted by *B. longum* has also been implicated in various processes. Recent research has confirmed that the EPS cluster is associated with *Bifidobacterium* biofilm formation, which plays an important role in adaptation to the intestinal environment.^31^ Similarly, Yan et al. found that *B. longum* extensively produces EPS, correlating with tolerance to artificial gastric and intestinal juices.^32^ However, the EPS produced by *B. longum* 105-A seemed to have the opposite effect. Tahoun et al. constructed a knockout mutant (ΔcpsD) that exhibited reduced polysaccharide production but stronger binding capacity to Caco-2 cells, suggesting a negative correlation between EPS production and the colonization ability of *B. longum* 105-A.^33^ It is well known that structure determines function. Therefore, the relationship between EPS and colonization may depend on the specific structure of the EPS produced by the bacteria to a great extent. In addition, studies have shown that oligosaccharides intake by the organism (e.g., raffinose and HMOs, etc.) can act as prebiotics to promote the colonization of *B. longum* in the intestine.^34^

## 3. Structure and composition of HMOs

**Figure 2.**
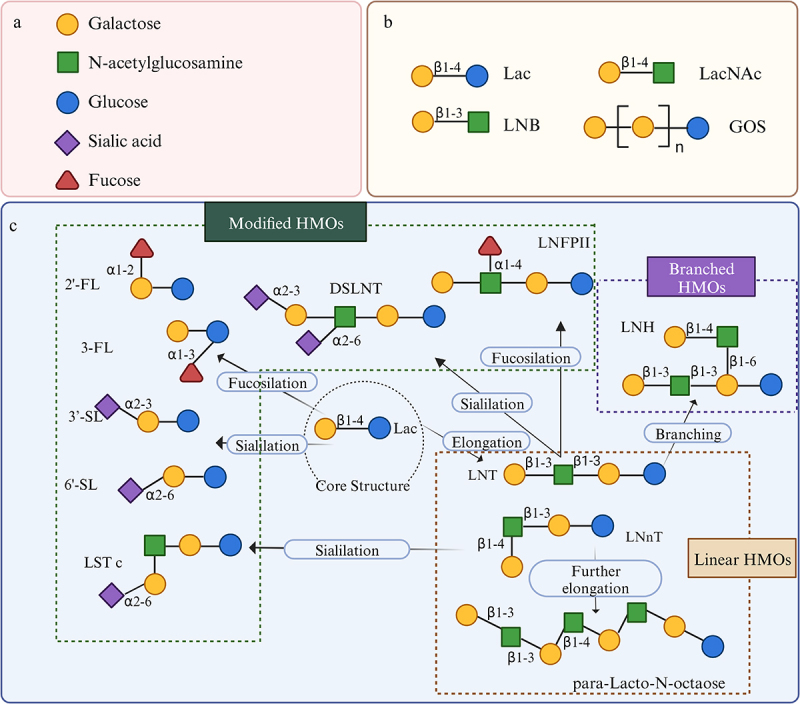
Structure of the main HMOs and GOS. (a) Five monosaccharides. (b) Disaccharide structure of HMOs and GOS. (C) HMOs with linear or branched structures modified by fucosylation or sialylation of the core lac structure. LNB, lacto-N-biose; LacNAc, N-acetyllactosamine; lac, lactose; HMOs, human milk oligosaccharides; 2’-FL; 2’-fucosyllactose; 3-FL, 3-fucosyllactose; LNnT, lacto-N-neotetraose; 3’-SL, 3’-sialyllactose; 6’-SL, 6’-sialyllactose; LNT, lacto-N-tetraose; LNFP II, lacto-N-fucopentaose II; LST c, sialyl-lnt c; LNH, lacto-N-hexaose; DSLNT, disialyllacto-N-tetraose.^35^ Image was drawn by Biorender.

Unlike Galactooligosaccharides (GOS), a substitute for HMOs, which are naturally found in breast milk and consist of glucose (Glc) and galactose (Gal). HMOs are generally composed of five monosaccharides: Glc, Gal, N-acetylglucosamine (GlcNAc), L-fucose (Fuc), and sialic acid (or N-acetylneuraminic acid, Neu5Ac). The diverse monosaccharide composition and glycosidic linkage contribute to the structural diversity of HMOs (Figure 2a,b).

HMOs are carbohydrates consisting of a lactose disaccharide covalently associated with one or more additional mono- or disaccharides. The core structure of HMOs is lactose (Lac) formed by linking Glc and Gal via a β-1,4-linkage. Lac can be modified by α-1,2/3 linkages to yield 2'-fucosyllactose (2'-FL) and 3-fucosyllactose (3-FL), classifying these as fucosylated HMOs (approximately 35–50% of all HMOs). Additionally, 3'-sialyllactose (3'-SL) and 6'-sialyllactose (6'-SL) are obtained by linking Neu5Ac through α-2,3/6 linkages, categorizing them as sialylated HMOs (approximately 12–14%).^36^ Further, lacto-N-biose (Galβ1-3GlcNAc-, LNB, type 1 chain) or N-acetylgalactosamine (Galβ1-4GlcNAc-, LacNAc, type 2 chain) can link through the reducing end of Gal via β1-3 to form lacto-N-tetraose (LNT) or lacto-N-neotetraose (LNnT), while LacNAc can link to LNT or LNnT via β1-6 to form lacto-N-hexaose (LNH) or Lacto-N-neohexaose (LNnH), respectively, and they are referred to as neutral non-fucosylated HMOs (about 42–55%). The mono- or disaccharide structure can be further extended to a linear or branching mode with one or more units, forming oligosaccharides with degrees of polymerization between 3 and 32, with type I chain HMOs being more abundant than type II chains^37^(Figure 2c).

In our previous study, a total of 31 breast milk samples from Jinan and Harbin, China, were analyzed for HMOs. The results revealed regional differences in HMO composition, with 2’-FL and LNT being the most abundant components, varying significantly across samples.^11^ This adequately proved the influence of geographical and individual differences of nursing mothers on HMO components. According to the secretor (*Se*)and Lewis (*Le*)blood-type status, nursing mothers are classified into four secretory types: *Se+Le+, Se+Le-, Se-Le+*, and *Se-Le-*. The ratio of these four types varies depending on the geopolitical situation or donor’s ethnicity.^38^ *Se* and *L*e encode α1-2-fucosyltransferase (FUT2) and α1-3/4-fucosyltransferase (FUT3), respectively. “Secretory mothers” with positive *Se* gene expression attach Fuc to the end of Gal via FUT2, enriching their breast milk with 2’-FL. Positive *Le* gene expression binds Fuc and Gal through FUT3, enriching breast milk with 3-FL and LNFP II, diversifying HMO composition among individuals.^39^ Simultaneously, it was shown that the effect of HMOs on intestinal structure may depend on their composition and their interaction with host microbes, which may be necessary for optimal intestinal adaptation.^40^

## 4. Utilization strategy of HMOs by *B. longum*

The complete breakdown of HMOs with different structures requires specific glycoside hydrolases (GHs) as well as transporters. *B. longum* possesses the largest and most varied array of transporters, GHs, catabolic, and transcriptional regulators involved in the metabolism of HMOs. Consequently, these bacteria can utilize a wide range of neutral, fucosylated and sialylated HMOs (Table 1). However, *B. longum* subsp. *infantis* and *B. longum* subsp. *longum* differ significantly in their ability and manner of utilizing HMOs. *B. longum* subsp. *infantis* is an avid consumer of HMOs and utilizes HMOs primarily in a “selfish” manner – completely transferring HMOs into the cell for degradation via specific transporters. *B. longum* subsp. *longum*, however, prefers plant-derived oligosaccharides over HMOs and has a limited and distinctly strain-specific ability to utilize HMOs, but can utilize HMOs both intra- and extracellularly.

**Table 1.** *Bifidobacterium longum* metabolizes HMO gene locus.

Protein Name	Locus Tag	Abbreviation	Origin	Refernce
**Extracellular glycosidases**				
Lacto-N-biosidase	BLLJ_1505	LnbX	*B. longum* ssp. *longum* JCM 1217	41
Chaperon for LnbX	BLLJ_1506	LnbY		
Transporters				
Type 1 HMO SBP	BLNG_00160	N.A.	*B. longum* ssp. *longum* SC596	42
Type 1 HMO SBP	BLNG_00936	N.A.		
FL transporter SBP	BLNG_01257	FL2-BP		
GNB/LNB transporter SBP	BLLJ_1626	GltA	*B. longum* ssp. *longum* JCM 1217	43
FL transporter SBP	Blon_0343	FL1-BP	*B. longum* ssp. *infantis* ATCC 15697	44
FL transporter SBP	Blon_2202	FL2-BP		
GNB/LNB transporter SBP	Blon_2177	GltA		
**Intracellular enzymes**				
α-1,2--Fucosidase	Blon_2335	AfcA	*B. longum* ssp. *infantis* ATCC 15697	45
α-1,3/4-Fucosidase	Blon_2336	AfcB		
α-2,3/6-Sialidase	Blon_2348	NanH2		46
LNT β-1,3-Galactosidase	Blon_2016	Bga42A		47
β-1,4-Galactosidase	Blon_2334	Bga2A		
β-N-Acetylglucosaminidase	Blon_0459	NahA		48
β-N-Acetylglucosaminidase	Blon_0732	Hex1		
β-N-Acetylglucosaminidase	Blon_2355	Hex2		
β-N-Acetylglucosaminidase	BLLJ_1391	N.A.	*B. longum* ssp. *longum* JCM1217	35
GNB/LNB phosphorylase	BLLJ_1623	LnpA		
Lacto-N-biosidase	BLLJ_1505	LnbX		41,49
Lacto-N-biosidase	BLLJ_1506	LnbX		
β-1,4-Galactosidase	BLNG_00015	Bga2A	*B. longum* ssp. *longum* SC596	42
LNT β-1,3-Galactosidase	BLNG_01753	Bga42A		
α-1,3/4--Fucosidase	BLNG_01263	AfcB		
α-1,2--Fucosidase	BLNG_01264	AfcA		
GNB/LNB phosphorylase	BLNG_00163	N.A.		

### 4.1. *Utilization strategy of HMOs by* B. longum *subsp.* infantis

*B. longum* subsp. *infantis* is one of the subspecies with the strongest ability to metabolize HMOs, and its HMO metabolic gene regions are conserved.^50^ Numerous studies have shown that the *B. longum* subsp. *infantis* grows robustly in media containing various HMOs, including 2’-FL, 3-FL, 3’-SL, 6’-SL, LNnT and LNT et al. as the sole carbon source (Table 2). As noted previously, *B. longum* subsp. *infantis* is presumed to metabolize HMOs intracellularly, and this “selfish” behavior of completely translocating HMOs into the intracellular space while limiting the sharing of metabolites with other species is the most efficient manner of utilizing HMOs, which can equally utilize type 1 and type 2 HMOs.^57^ Similarly, the glycoprofile analysis of HMO consumption showed that *B. longum* subsp. *infantis* exhibits a preference for HMOs with a degree of polymerization ≤7.^58^ This may be attributed to the transporter’s number, which impedes the metabolic uptake of high molecular weight HMOs into the cell, thus inhibiting their intracellular translocation.^59^

**Table 2.** Degradation of HMOs by *B. longum.*

Strain	2′-FL	3-FL	3′-SL	6′-SL	LNnT	LNT	Reference
*B. longum* subsp. *infantis*							
ATCC15697	+	+	+	+	+	+	51,52
M-63	+	+	+	+	+		51
DSM20088	+	+	–	–	+		53
BRS8-2	+	+	+	–	+		
TPY12-1	+	+	+	–	+		
Y538	+	+			+	+	54
ATCC 15702	+	+		+	+	+	55
JCM 7011	+	+		+	+	+	
SC142	+	+		+	+	+	
*B. longum* subsp. *longum*							
SC596	+	+	–	–	+	+	42
SC558	–	+	–	–	+	+	
SC618	–	–	–	–	+	+	
JCM11347	+	+	+	+			56
JCM7010	+	+	+	+			

Plus (+) indicates degradation of HMOs tested, minus (−) no degradation.

**Figure 3.**
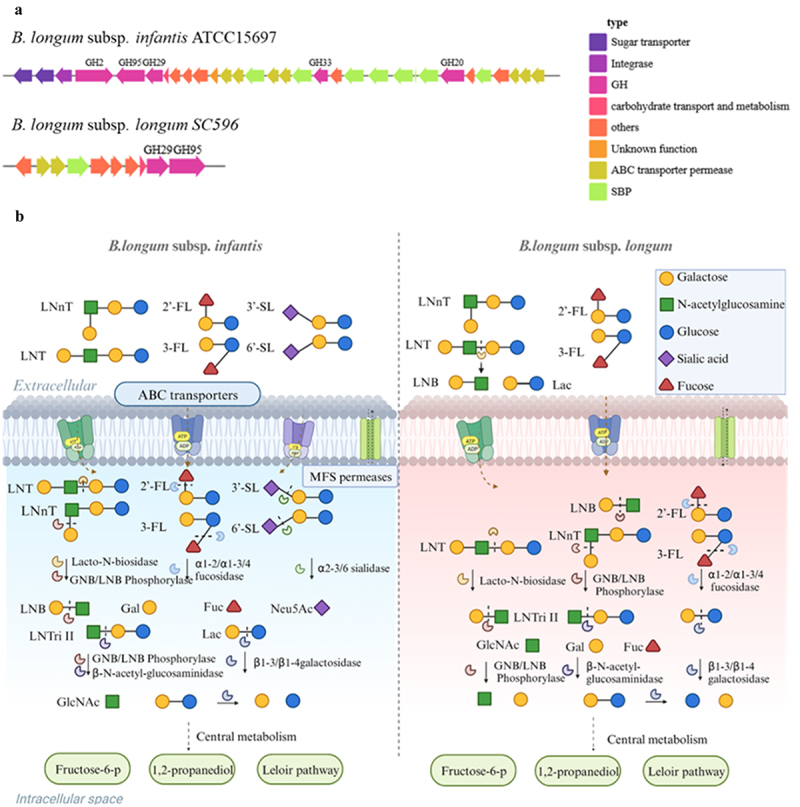
Utilization strategy of HMOs by *B. longum* (a) Representative *B. longum* metabolizes HMOs gene clusters (b) Metabolic processes of major HMOs in *B. longum*. LNT: lacto-N-tetraose; LNnT: lacto-N-neotetraose; LNB: lacto-N-biose; 2’-FL: 2’-fucosyllactose; 3-FL: 3-fucosyllactose; 3’-SL: 3’-sialyllactose; 6’-SL: 6’-sialyllactose. Image was drawn by Biorender.

In a previous study, our lab performed whole-gene sequencing of *B. longum* subsp. *infantis* E4 and found that it is enriched in the GH family (GH1, GH2, GH13, GH4, and GH38), which confers specific carbohydrate metabolism and transport capabilities.^60^ Meanwhile, genomic sequence analysis of *B. longum* subsp. *infantis* ATCC15697 revealed a 43 kb gene cluster Ι (Figure 3a).^61^ This cluster encodes intracellular GHs, ATP-binding cassette-type (ABC) transporters, and extracellular solute-binding proteins (SBPs). The SBPs exhibit affinity for a variety of specific HMOs and are members of the ABC transporter superfamily of oligosaccharides.^62^ *B. longum* subsp. *infantis* encodes ABC transporters for the transfer of intact HMOs into the cytoplasm, mediated by family 1 SBPs. Once inside the cytoplasm, a cascade of cytoplasmic GHs converts various HMO molecules into monosaccharides. The entire hydrolysis process starts with the removal of Fuc and Neu5Ac by α-l, 2-fucosidase of the GH95 family or α-l, 3/1-4-fucosidase of the GH29 family and α-2,3/6-sialidase of the GH33 family.^46^ GNB/LNB is reversibly phosphorylated by GNB/LNB phosphorylase of the GH112 family. Moreover, β-N-acetyl-glucosaminidase from the GH20 family can release GlcNAc.^63^

The core structure of HMOs is exposed upon removal of the modifying moiety, leading to further disassembly by the two galactosidases. GH2 family β1,4-galactosidases tend to use Lac and LNnT, while GH42 family LNT β-1,3-galactosidases were found to prefer type I HMOs. Research has shown that the specific activity of β1,4-galactosidases toward LNT was only 0.005% of that of LNT β-1,3-galactosidases.^64^ The resulting GAL and GLC are metabolized by the Leloir and fructose-6-phosphate (F6P) phosphoketolase pathways (Figure 3b).^65^

### 4.2. *Utilization strategy of HMOs by* B. longum *subsp.* longum

Although *B. longum* subsp. *longum* belongs to the same species as *B. longum* subsp. *infantis*, its genome contains smaller number of GHs and transporter proteins capable of utilizing HMOs. It employs an extracellular utilization strategy for certain HMOs, albeit infrequently (Table 1). Previous studies have discovered that strains of *B. longum* subsp. *longum* contain extracellular GH136 lacto-N-biosidase (LnbX), which requires a specific chaperone (LnbY) for proper folding, LnbX-positive expression strains take up lacto-N-biose (LNB) using GNB/LNB-BP (GltA) and use GNB/LNB phosphorylase (LnpA) for subsequent intracellular phosphorolysis. In contrast, LnbX-negative expression strains employ LNT β-1,3-galactosidase to break down LNT within the cells, after which the intracellular metabolic pathway, similar to that of *B. longum* subsp. *infantis* decomposed the substrate into monosaccharides (Figure 3b).^66^ This is consistent with findings by Gerben et al. that 61 strains of *B. longum* subsp. *longum*, either isolated from adults or infants, grew well on LNT, but none could grow on 2’-FL, 3-FL, 3’-SL, and 6’-SL.^67^

However, studies have demonstrated that specific strains of *B. longum* subsp. *longum* possess the ability to utilize other HMOs (Table 2). For instance, Garrido et al. discovered that *B. longum* subsp. *longum* SC596 can grow vigorously on 2’-FL and 3-FL. By analyzing the strain’s genome, Garrido et al. identified a novel cluster of genes devoted to fucosylated HMO utilization (FHMO), which encompassed genes for the transportation of fucosylated molecules, fucose metabolism and ⍺-fucosidases that belong to GH29 and GH95 families, respectively (Figure 2a). FHMO confers the unique ability to preferentially consume fucosylated HMOs to *B. longum* subsp. *longum* SC596.^42^ Most notably, the number of *B. longum* subsp. *longum* that can utilize fucosylated HMOs is limited, and the prevalence of the FHMO gene cluster in *B. longum* subsp. *longum* is less than 3%.^48^ Some of the *B. longum* subsp. *longum* that can grow on FHMO have recently been renamed “transitional *B. longum*”.^68^ This reclassification indicates evolving knowledge of HMO-utilizing genes, suggesting that further in-depth research on the genetic mechanisms related to HMOs utilization is required.

### 4.3. *Cross-feeding of HMOs in* B. longum

The breakdown products of HMOs can serve as growth substrate for other microorganisms, regulating the intestinal microbiota through a process known as cross-feeding.^69^ One of the key rationales for the dominance and ubiquity of *Bifidobacterium* in the early microbiota is that they may access polysaccharides or monosaccharides in the intestine through reciprocal cross-feeding. For instance, *B. longum* subsp. *infantis* can dominate by prioritizing the utilization of HMOs through ecological niche grabbing, whereas *B. breve* has a limited ability to utilize HMOs but benefits from the priority effect (the preferentially colonized *B. longum* subsp. *infantis*, provide *B. breve* with fucose and other degradates that contribute to its dominance).^70^ Collaboration with other HMO or non-HMO consumers for the degradation and metabolism of HMOs may help to increase the diversity and dominance of *Bifidobacterium* .^71^

Since *B. longum* subsp. *infantis* transfers HMOs into cells for degradation, this “selfish” behavior prevents it from sharing the intermediates of HMOs metabolism with other intestinal bacteria. However, it can use the intermediates produced by other intestinal bacteria to promote its own growth. For instance, a cross-feeding mechanism was found in the degradation of HMOs between *B. bifidum* JCM1254 and *B. longum* 150-A, JCM1254 has a complete HMOs degradation gene cluster, and 150-A only grew well on HMOs when co-cultured with JCM1254, suggesting that catabolic products of JCM1254 could be utilized by 150-A.^72^ Cheng et al. conducted a study co-culturing *B. longum* subsp. *infantis* with *F. prausnitzii* using different HMOs (2’-FL, 3-FL, 6’-SL, and LNT2) as the sole carbon source. The results showed that compared to monoculture, co-culture not only promoted acetate production but also increased the utilization of 6’-SL. However, this phenomenon was not observed in cultures with other HMOs. They hypothesized that co-culture may enhance sialidase expression, promoting the growth of *B. longum* subsp. *infantis*, and the utilization strategy of HMOs may highly depend on their structure.^73^

*B. longum*, in turn, metabolizes HMOs into monosaccharides and enters the ‘bifidobacterial pathway’, producing products that promote the growth of other *Bifidobacterium*. For example, lactose produced by *B. longum* LH206 after degrading LNnT can be used as a carbon source to promote the growth of *B. pseudocatenulatum* LH663 (non-LNnT degrader), which also resulted in an increase in acetate, ethanol, and formate after the growth of LH663 in the supernatant of LH206 utilizing LNnT.^71^ In fact, other non-bifidobacterial species may also benefit from the cross-feeding mechanism. Chia et al. demonstrated that *Anaerostipes caccae*, which is unable to use HMOs, can grow with *B. longum* subsp. *infantis* ATCC15697 in a medium in which HMOs were the sole carbon source. ATCC15697 metabolized HMOs to produce monosaccharides as a carbon source for the growth of *Anaerostipes caccae*, which also synthesized butyric acid using lactic acid and acetic acid produced by the metabolism of HMOs by ATCC15697 as precursors. This cross-feeding process provides a pathway for intestinal enrichment of the intended metabolites.^74^

## 5. Effects of HMOs and *B. longum* in infants healthy

### 5.1. Promote the development of intestinal immune system

At birth, the neonatal immune system is structurally and functionally immature, making infants less capable of preventing and clearing pathogenic infections compared to adults. The neonatal immune system is biased toward humoral immunity of the TH2 type.^75^ Daily supplementation of 2’-FL to healthy newborn rats resulted not only in a significant increase in plasma IgG concentration and an increase in the ratio of Th1/Th2 immunoglobulins (IgG 2b + IgG 2c/IgG 1 + IgG 2a), suggesting that supplementation with 2‘-FL promotes a shift of the immune system toward a TH1 response. *B. longum* has also been found to have the same ability. *B. longum* subsp. *infantis* GB-1496, isolated from human milk, showed a strong immunomodulatory capacity, as evidenced by higher induction of Th1-associated cytokines and lower production of Th2-associated cytokines, effectively switching the immune response from Th2 to Th1 polarization.^76^

Furthermore, in a previous study of our lab, maternal feeding of 2′-FL resulted in significantly higher spleen and thymus indices and lymphocyte proliferative capacity in offspring mice. Interestingly, maternal feeding of 2′-FL in combination with *Bifidobacterium* resulted in significantly higher serum levels of IgG in the offspring. IgG is considered the only maternally available antigen that can cross the placental barrier and be secreted and delivered by the mammary glands, leading to the speculation that there is a “vertical enhancement pathway” for the immune system. It means maternal supplementation with HMOs and *Bifidobacterium* together, which may promote the maturation of the offspring’s immune system.^77^

The neonatal intestine needs to develop rapidly to support growth, and intestinal stem cells, which are found in the crypts of the intestine, are essential for the continual renewal of the intestinal epithelium to maintain nutrition absorption and immune function. Fucose, as a constituent of HMOs, has been shown to increase the number of goblet cells in the mouse intestine, promoting intestinal epithelial differentiation, supporting intestinal organoid outgrowth and enhancing stem cell stemness in vitro.^78^ Wang et al. found that HMOs derived from human milk promoted the maturation of crypt-like organs and increased the number of Ki67-positive cells, a marker of cell proliferation.^79^ In addition, Paneth cells play an important role in the innate immunity of the small intestine and in the proliferation of intestinal stem cells. Zhou et al. proved that the function of Paneth cells in maintaining the stem niche is restored by *B. longum*. However, in this experiment, the closed structure of the intestinal organoids limited the interaction with *B. longum*, leading researchers to hypothesize that soluble small molecules secreted by *B. longum* may play a direct role. Combined with our previous findings that the ability of strains to produce short-chain fatty acids was positively correlated with the ability to promote LGR5 (intestinal stem cell marker) expression.^80^ We hypothesize that HMOs and *B. longum* may also promote intestinal development through the production of metabolites, though this also needs to be confirmed through extensive animal and clinical studies.

### 5.2. Prevention of susceptible diseases

#### 5.2.1. Prevention of necrotising enterocolitis (NEC)

Necrotising enterocolitis (NEC) is a catastrophic intestinal disease that primarily affects premature infants. Andrea et al. performed HMOs profiling of breast milk fed to infants with NEC and metagenomic sequencing analysis of feces from these infants. They found that a single HMO, disialyllacto-N-tetraose, was lower in this breast milk, consistent with the finding by Wejryd et al., who noted that a low diversity of HMOs is associated with NEC. Correspondingly, *B. longum* abundance in the feces of infants with NEC was significantly lower than that of the control group. This study provides a new method for targeted mitigation of NEC by demonstrating the importance of HMOs and *B. longum* in preterm infants.^81,82^ In addition, HMOs and *Bifidobacterium* have been shown to have certain effects in preventing NEC in numerous research (Table 3).

**Table 3.** Human milk oligosaccharides and *Bifidobacterium longum* in the prevention of intestinal diseases in research.

Intestinal disease	Type of experiment	Object	Intervention	Conclusion	Reference
Necrotising enterocolitis (NEC)	clinical experiment	infants weighed <1500 g at birth; received full resuscitation and survived until day of life 3.	Daily feeding *+B. longum* subsp. *infantis* EVC001(8 × 10^9^ CFU/d).	The cumulative incidence of NEC in the EVC001 group was reduced from 11% to 2.7%, a 73% risk reduction, and the mortality rate associated with NEC was reduced to zero.	83
		preterm infants with a birth weight of <1500 g in three Austrian neonatal intensive care units where different regimens for the prevention of NEC were used.	human milk + *Lactobacillus rhamnosus* LCR 35+ nystatin+enteral gentamicin; formula milk + *Bifidobacterium longum* subsp. *infantis* NCDO 2203 and *Lactobacillus acidophilus* NCDO 1748+ fluconazole; human milk + enteral gentamicin+ nystatin.	The beneficial effects of *Bifidobacterium longum* subsp. *infantis* NCDO 2203 supplementation depends on simultaneous feeding with HMOs, which may be a promising synergistic approach to NEC.	84
	animal experiment	7–8-day neonatal mice: treated with milk formula containing stool slurry collected from children with severe NEC (12.5 μL/mL) five times a day, then exposed to hypoxia for 10 min twice daily for 4 days.	NEC + lactose (10 mg/mL); +2’-FL (10 mg/mL); +6’-SL (10 mg/mL); +2’-FL + 6’-SL (5 mg/mL).	The addition of 2’−FL and 6’-SL alone or in combination can exert anti-NEC effects, and 2’−FL and 6’-SL, but not lactose, dock into the binding pocket of the TLR4-MD2 complex, inhibiting TLR4 signaling.	85
		7–10-day mice: Similac Advance infant formula: Esbilac dog milk replacer at a 2:1 ratio at a dose of 50 µL/g body weight, supplemented with enteric bacteria from an infant with severe NEC 5 times/day.	NEC +2’-FL (5 mg/mL of formula, 0.25 mg/g body weight, once daily).	2’-FL attenuates NEC severity through upregulation of the vasodilatory molecule endothelial nitric oxide synthase, increasing neonatal intestinal mesenteric perfusion.	86
Infection	clinical experiment	Healthy-term newborns (≥37 weeks), with normal birth weight (3rd to 97th percentiles forgestational age), and ≤3 months of age at enrollment.	formula milk + *Bifidobacterium longum* subsp *infantis* CECT7210(10^7^ CFU/g).	Administration of *Bifidobacterium longum* subsp *infantis* CECT7210 reduced the frequency of diarrhea in infants compared to without the addition of CECT7210.	87
		Healthy, full-term male and female infants of 0 to 14 days of age	+2-HMO formula milk (1.5 g/L lactose was replaced by a 2:1 mixture of two HMOs, respectively, 2′-FL and LNnT at 1.5 g/L)	HMO consumption increased the concentration of glutamine amidated amino acids (GGAA), which was negatively correlated with lower respiratory tract infections, and *B. longum* may drive the metabolic pathway of GGAA.	88
	animal experiment	BALB/c mice weighing 10–20 g: oral challenge with 10^4^ or 10^8^ CFU/mL of *Campylobacter jejuni* 287ip.	mouse model +2 mg/mL neutral milk oligosaccharides (total of 6 mg in three separate supplements).	An H-2 carbohydrate moiety, perhaps the essential component of HMOs, acts as a soluble receptor for *Campylobacter jejuni*, preventing it from binding to its intestinal receptors and protecting infants from enteric pathogens.	89
		Wild-type C57BL/6J female mice, aged 7 weeks: inoculated with 1 × 10^9^ CFU/mL Group B *Streptococcus* (GBS) COH1 into the vaginal tract	mouse model +100 mg/mL pooled HMOs isolated from human milk (pHMOs); +100 mg/mL LNT.	Treatment with pHMOs reduces the burden of GBS in the vagina in vivo with minimal changes in the vaginal microbiota, and the effect of a single LNT is not significant. Mixed HMOs are a promising therapeutic bioactive agent to limit the colonization of the vagina by GBS.	84
Food allergy	clinical experiment	Milk samples were collected from mothers participating in a high-risk allergy birth cohort and clinical skin prick tests were performed on their infants at 0.5, 2, 12 and 18 years of age.	No additional intervention	Children exposed to acidic predominant HMOs may be protected from food sensitization.	90
	animal experiment	8–9-week-old adult male BALB/c mice (20-25 g): treated on days 0 and 14 with 50 µg ovalbumin (OVA) and 2 µg alum in 200 µl PBS, and on day 28 and thereafter with 50 µg OVA in PBS every 3 days (six times).	OVA-sensitized mice + 2’-FL (1 mg/200 μL); + 6’-SL (1 mg/200 μL); +lactose (1 mg/200 μL).	2’-FL and 6’-SL induce IL-10+ T regulatory cells and indirectly stabilize mast cells to reduce symptoms of food allergy.	91
		Female BALB/c mice (5–6 weeks, 15–20 g): injected with 0.2 mL of 1 mg/mL β-lactoglobulin (β-LG) dissolved in a mixture of Freund’s adjuvant on d 7, 14, and21.	β-LG-sensitized mice + HMO (400 mg/kg); + 2′-FL (200 mg/kg); + 2’-FL (400 mg/kg); + 2’-FL (600 mg/kg).	Oral administration of 2′-FL or HMOs to food-allergic mice reduces β-LG-induced serum-specific IgE secretion and mast cell degranulation and decreases secretion of inflammatory factors, thereby alleviating food allergy symptoms.	92
		6-8 weeks BALB/c wild-type mice: injected with OVA (50 mg) with aluminum potassium sulfate adjuvant (1 mg) once and then again 2 weeks later. Two weeks after the injections, OVA (10 or 50 mg) was administered orally every other day.	OVA-sensitized mice + *B longum* KACC 91563 (5 × 10^9^ CFU/mouse); + *Enterococcus faecalis* KACC 91532 (5 × 10^9^ CFU/mouse)	*B. longum* KACC 91563 to OVA-sensitized mice showed that this strain and its derived extracellular vesicles could attenuate the allergic response by selectively inducing apoptosis of mast cells, the main effector cells in food allergy.	93
		Female BALB/c mice aged 8 weeks: injected on day 1 and day 14 for OVA sensitization with 100 μg OVA and 1 mg alum as adjuvant.	OVA-sensitized mice + *B. longum* CECT7894 (2 × 10^8^ CFU/d)	*B. longum* CECT7894 modulates sphingolipid metabolism and reduces IgE levels, and high levels of IgE cross-linked to FcεRIs can lead to mast cell activation and food allergy symptoms.	94
		3-week-old female BALB/c mice: sensitized by intragastric gavage with β-LG (1 mg/g body weight) plus cholera toxin (10 μg/mouse) as an adjuvant.	β-LG-sensitized mice + *B. longum* BBMN68 (2 × 10^9^ CFU/d)	*B. longum* BBMN68 reduced allergic reactions by stimulating dendritic cells to increase the number of regulatory T cells.	95

However, Melendez et al. did not find HMOs and *B. longum* to be protective in NEC. They added *B. longum* subsp. *infantis* and 3’-SL alone or in combination with the daily formula-based diet and found that the severity of the NEC model disease was negatively correlated with the abundance of *B. longum* subsp. *infantis* in the intestinal contents. Although the addition or absence of both could slightly reduce the incidence of NEC, there was no significant difference from the control group. Interestingly, a significant decrease in the incidence of NEC was found in the group fed human milk.^96^ Therefore, we speculated that it is not only the specificity of the strains that leads to the difference in effect on NEC but also the effect of HMOs with different structures on NEC.

#### 5.2.2. Prevention of infection

Diarrhea is the leading cause of morbidity and mortality in children under five in low-income countries, with pathogenic bacterial infections being the primary cause of neonatal diarrhea.^97^ HMOs may prevent diarrhea by reducing the likelihood of infection by pathogenic bacteria, both indirectly and directly. On the one hand, HMOs act as soluble decoy receptors that resemble glycans on the mucosal cell surface of pathogenic bacteria, which can directly inhibit the binding of pathogenic bacteria to intestinal epithelial cells. However, this may be related to the type of human milk, as studies have shown that human milk from secreting mothers may prevent the attachment of recombinant norovirus particles to their primary receptors compared to human milk from non-secreting mothers, thereby shielding infants from norovirus infection.^98^ On the other hand, HMOs can also directly bind to the intestinal epithelial cells, which can play a competitive inhibitory role with the pathogenic bacteria.^99^

Although HMOs and *B. longum* have each been shown to be effective in preventing diarrhea in infants, no studies have evaluated their function in combination, necessitating further research to determine if their synergistic effect enhances diarrhea prevention. In addition, they have also been shown to play a positive role in the prevention of lower respiratory tract infections and Group B *Streptococcus* infections (Table 3).

#### 5.2.3. Prevention of food allergy

As the gut microbiota and immune system of infants are constantly changing in the early stages of life, this is a susceptible period for food allergies.^100^ Evidence suggests that HMOs and *B. longum* may have a potential role in the prevention of allergies (Table 3). However, Miliku et al. concluded that individual HMOs did not have an effect on food allergy prevention; instead, a combination of HMOs is effective.^101^ This finding highlights the importance of using a mixture of HMOs rather than a single type to prevent food allergies.

Although a synergistic study of HMOs with *B. longum* in food allergy is not currently available, studies have shown that the development of food allergy is associated with intestinal inflammation. In a macro-genomic analysis of 208 infants, the absence of *Bifidobacterium* and genes for HMO utilization in the intestinal tract was linked to immune imbalance and induced intestinal inflammation. Conversely, supplementation with a strain of *B. longum* subsp. *infantis* EVC001, which has high HMO utilization genes, favored TH1 polarization and reduced inflammation by regulating TH2 and TH17, key components of allergic susceptibility.^100,102^ In conclusion, the use of HMOs as prebiotics for *B. longum* seems to have potential applications in the prevention of food allergy.

## 6. Protective mechanisms of HMOs and *B. longum* in infants

### 6.1. Promote the production of beneficial metabolites

Interactions between microbial metabolites and the host are one of the mechanisms by which gut microbiota exert beneficial effects. Dietary indigestible carbohydrates (e.g. HMOs in breast milk) are fermented in the gut to produce various beneficial metabolites, such as short-chain fatty acids (SCFAs) and aromatic lactic acid etc.^103^

SCFAs significantly connect the microbiota to the host’s immune system. *B. longum* metabolizes HMOs to produce acetic acid, in the meanwhile, the addition of HMOs (2’-FL, LNnT, 3’-SL, and 6’-SL) to feces for fermentation has also been shown to differentially elevate acetic and propionic acid content. Although butyric acid content was not significantly elevated, it was shown that HMOs specifically enrich butyrate producers.^104,105^ Acetic acid, propionic acid, and butyric acid are commonly present in the colon and can be absorbed by the intestinal epithelial cells, decreasing the intestinal pH and inhibiting pathogenic bacteria growth.^106^ They bind to G protein-coupled receptors (GPCRs) and hinder the action of Histone deacetylases (HDACs), thereby altering cell differentiation, proliferation, downstream inflammation regulation, and other signaling pathways, affecting cell differentiation, proliferation, and apoptosis, etc.^3^ In the study by Salli et al. a semi-continuous colon simulator was applied to explore the changes in metabolites of 2’-FL and 3-FL during co-fermentation with *B. longum* subsp. *infantis* Bi-26. The results showed that the incorporation of HMOs significantly elevated the overall content of SCFAs, in particular the content of acetic acid.^107^ Tsukuda et al. sequenced feces collected from breastfed infants and found that an increase in *B. longum* subsp. *infantis* abundance correlated with increased formic acid concentration, and *B. longum* subsp. *infantis* IN-F29 was able to produce formic acid through HMO-derived fucose.^108^

Laursen et al. identified a previously unknown aromatic lactate dehydrogenase in *Bifidobacterium* that metabolizes tryptophan, phenylalanine and tyrosine to form aromatic lactic acids (indolyllactic acid (ILA), phenyllactic acid (PLA), and 4-hydroxyphenyllactic acid (4-OH-PLA)). Analyzing infant fecal samples revealed that the abundance of these aromatic lactic acids was positively correlated with the *Bifidobacterium* species. Further mechanistic investigation of the whole process revealed that *Bifidobacterium*-derived ILA is highly associated with activation of the AhR and HCA 3 pathways, reducing inflammation and protecting against pathogens.^109^ Another study also found a high level of *B. longum* subsp. *infantis* correlated with high ILA abundance and compared to lactose as a growth substrate, *B. longum* subsp. *infantis* grown on HMOs produced more ILA. ILA increased mRNA expression of CYP1A1, the target gene of the AHR, reducing the activation of downstream inflammatory pathways and the secretion of pro-inflammatory factors.^110^

### 6.2. Improved intestinal barrier

**Figure 4.**
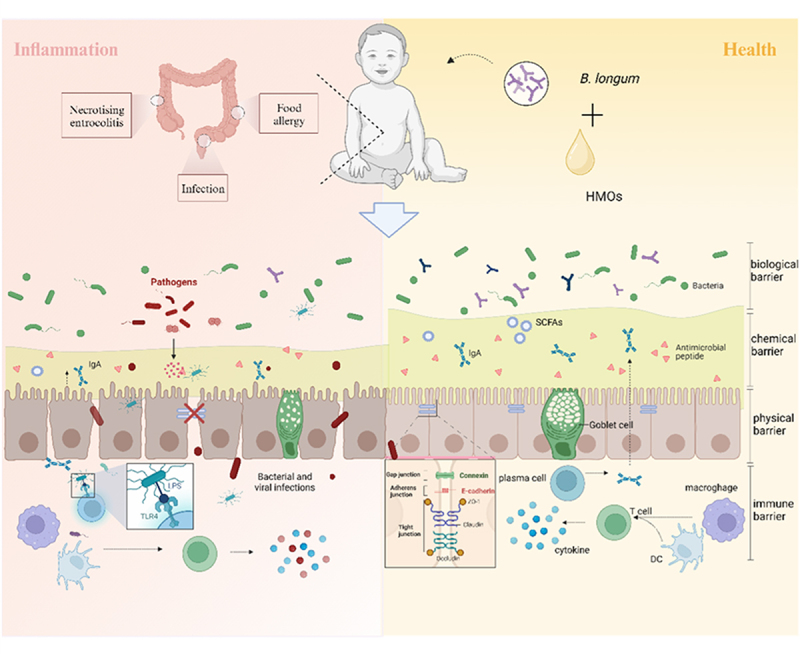
Mechanism by which *B. longum* and HMOs work together to protect the intestinal barrier. SCFAs: short chain fatty acids; IgA: immunoglobulin A; DC: dendritic cell. Image was drawn by Biorender.

The intestinal barrier is the gatekeeper of the human body that prevents harmful substances (eg. bacteria and various toxins, etc.) from passing through the intestinal mucosa into the bloodstream and other organs. It can be divided into four different layers of sub-barriers: biological barrier, chemical barrier, physical barrier, and immune barrier (Figure 4). Disruption of the intestinal barrier allows intestinal microorganisms and toxins to enter the bloodstream, leading to systemic inflammation.^111^ Therefore, reinforcing the intestinal barrier is crucial for infants with weaker immune systems.

#### 6.2.1. Enhanced biological barrier

Biological barriers are complex ecosystems formed by hosts and parasitic microorganisms. A rich and diverse microbiota maintains intestinal homeostasis through symbiosis, antagonism, and competition for binding sites with pathogenic bacteria.^112^ With the in-depth study of intestinal microbiota, it is debatable whether changes in microbiota are a cause or a consequence of disease pathogenesis, but there is no doubt that disrupted intestinal microbiota can transmit disease as demonstrated by several fecal microbiota transplantation (FMT) experiments, suggesting that a healthy intestinal microbiota is essential.^113^

Evidence suggests that HMOs and *B. longum* individually benefit the biological barrier by regulating gut microbiota. Jane et al. found that 2’-FL, 2’-FL/LNnT and a mixture consisting of six HMOs all increased the relative abundance of Actinobacteria and decreased the abundance of Firmicutes, compared with the addition of lactose to a microbiological mimicry system for infants’ gut. Whereas different HMOs affected specific families, for example, 2’-FL specifically increased the levels of Bifidobacteriaceae and Coreobacteriaceae, and 2’-FL/LNnT increased the levels of Brucellaceae. Mixtures of HMOs increased the relative abundance of Ruminalococcaceae, which have been shown to be correlated with butyric acid production.^114^ Similarly, *B. longum* could increase the abundance of potential probiotics such as *Akkermansia muciniphila* and restore intestinal homeostasis by regulating the ratio of Firmicutes/Bacteroidetes^115^

When applied as synbiotics, *B. longum* increases its colonization capacity due to its efficient HMOs utilization. For instance, the application of synbiotics to antibiotic-induced dysbiotic microbiota revealed that synergism with HMOs is essential for the persistent colonization of *B. longum* subsp. *infantis*. Additional synbiotics guided microbiota restoration to a more coherent community structure, increasing *B. longum* abundance and that of *Lactobacillus* and *Veillonella*, which utilize lactic acid from synbiotic metabolism to produce propionic acid via cross-feeding.^116^ Another experiment similarly showed lower HMO concentrations in samples supplemented with *B. longum* subsp. *infantis* EVC001 compared to the fecal composition of breastfed infants not supplemented with *B. longum* subsp. *infantis* EVC001, suggesting that depletion of HMOs increased the ability of *B. longum* subsp. *infantis* EVC001 to colonize the fecal composition of breastfed infants and that this effect persisted after cessation of supplementation, together with a reduction in the abundance of gram-negative Proteobacteria and Bacteroidetes and a fourfold reduction in endotoxin levels.^117^ *B. longum* directly metabolizes acetic acid produced by HMOs or indirectly increases the abundance of strains producing different SCFAs, further acting on other sub-barriers to establish a healthy intestinal barrier.

#### 6.2.2. Enhanced chemical barrier

The chemical barrier includes water in the intestinal cavity, digestive fluid, and the intestinal mucus layer, which protects the intestinal mucosa from harmful microorganisms and acid and alkali erosion, providing complete defense for epithelial cells against harmful substances and pathogens. Mucin2 (MUC2) is the major secreted mucin in the small and large intestine that secretes Cathelicidins-associated antimicrobial peptides, and in MUC2-deficient mice bacteria interact directly with the intestinal epithelium to induce inflammation due to the lack of protection from the mucus layer.^118^

Richard et al. applied HMOs to cells and organoids in vitro as well as in the NEC mouse model, demonstrating that HMOs increased goblet cell numbers, enhanced endoplasmic reticulum folding through regulation of endoplasmic reticulum chaperone proteins, and thus facilitated MUC2 secretion.^119^ Likewise, supplementation of 2′-FL in the daily diet significantly increased the expression of the IL-22 gene in the ileum of model mice, a key cytokine regulating epithelial homeostasis, increasing goblet cell numbers, promoting mucin secretion, and decreasing intestinal permeability.^120^

Mucins have a similar structure to HMOs and can be degraded by some bacteria, and a high abundance of mucus-degrading bacteria can damage the intestinal mucus barrier. *B. longum* subsp. *infantis* is unable to utilize mucins due to the lack of transporters. In the intestines of breastfed infants highly colonized with *B. longum* subsp. *infantis* EVC001, colonization with EVC001 was found to reduce mucinoglycan utilization in the colon, protecting the intestinal barrier.^121^ Interestingly, *Akkermansia muciniphila* uses mucin as an energy source, but numerous experiments have confirmed its positive regulatory effects on intestinal mucus layer thickness and intestinal barrier integrity.^122^ Why are some mucin-degrading bacteria beneficial to mucus maintenance and others detrimental? The mechanisms of action of different mucin-degrading bacteria need to be further explored!

After pretreatment with the combination of 2’-FL and *B. longum* subsp. *infantis*, the number of MUC2-positive cells in the ileum and colon was significantly higher than in the control group.^123^ To further explore the components promoting mucin secretion, in addition to the role of the bacteria’s role, products of their synergistic metabolism may also play an important role. For instance, it has been shown that mucus-associated bacteria promote mucus secretion and increase mucus layer thickness by releasing microbe-associated molecular patterns (MAMPs) and producing SCFAs.^122^ Specific metabolites targeting the chemical barrier aspects of HMO interactions with *B. longum* subsp. *infantis* remain to be confirmed in further experiments.

#### 6.2.3. Enhanced physical barrier

The physical barrier consists mainly of connections between intestinal epithelial cells and neighboring cells, forming an intact intestinal epithelial structure. The permeability of the lateral space between neighboring epithelial cells (known as the paracellular space) is controlled by the apical tight junctions (TJs), which consist of complexes of cytoplasmic and membrane-associated proteins and are the structural foundation of the physical barrier of the intestinal mucosa.^124^ The epithelial junction component of the mechanical barrier can be detected during the development of the infant in utero but does not play a role until full-term delivery, and then the infant’s postnatal exposure to a new microbiota can have an impact on the TJ protein composition, altering the intestinal physical barrier permeability.^125^

A mixture of six HMOs (2’-FL, difucosyllactose, LNT, LNnT, 3’-SL and 6’-SL) acting on cytokine-induced inflammatory cells was able to modulate in a dose-dependent manner fluorescein-isothiocyanate (FITC)-labeled Dextran 4 KDa (FD4) translocation and increased TEER values, which reflect the integrity of cellular tight junctions. Moreover, it was found that 2’-FL contributed significantly to the enhancement of the intestinal barrier.^126^ Similar results were obtained by Kim et al. who applied 2’-FL and 3-FL to Caco-2 cells as well as to DSS mice, finding that both decreased FD-4 permeability, increased ZO-1 and Occludin protein levels, and decreased Claudin-2 protein expression, both in vitro and in vivo.^127^ Similarly, *B. longum* has also been shown to have a protective effect against physical barriers. Chen et al. found that the expression of TJ proteins ZO-1, Occludin-1, and Claudin-1 was significantly elevated in the colons of mice after administration of *B. longum* HB5502 by gavage to TNBS-induced IBD model mice.^128^

*B. longum* subsp. *infantis* grown utilizing HMOs increased the expression of adhesion molecules (AM) in Caco-2 cells and ZO-1 in HT-29 cells and prevented the delocalization of Occludin to the cytoplasm. Redistribution of TJ proteins from intercellular junctions to intracellular compartments may suggest a defect in the barrier function.^129^ Additionally, as mentioned earlier, *B. longum* can use HMOs to produce ILA as a ligand for AHR, the activation of which is closely linked to the expression of TJ proteins. The stimulation of acetate production by *B. longum* metabolism on LNT and LNnT specifically inhibited *Salmonella* invasion, which also improved the integrity of the intestinal barrier.

#### 6.2.4. Enhanced immune barrier

The immune barrier is composed of gut-associated lymphatic tissue (GALT) and secretory immunoglobulin A (SIgA), along with the corresponding immune response. This barrier is crucial for maintaining intestinal immune homeostasis. Early microbial colonization is essential for the development of the immune system, and the absence of microbial colonization results in defective immune system development and low numbers of immune cells.^130^ *B. longum*, as one of the earliest intestinal colonizers, and HMOs, as “bifidus-factors”, both of which, together with their metabolites, significantly enhance the immune barrier.

Our lab previous studies have shown that additional supplementation of 2’-FL in infant formula enriched *Bifidobacterium* in the intestinal tract and achieved immune function similar to breastfeeding by up-regulating the levels of the anti-inflammatory cytokines IL-2, IL-9, and IL-10, while down-regulating the levels of the pro-inflammatory cytokine IL-4.^131^ Eiwegger et al. exposed monocytes derived from umbilical cord blood to a mixture of saliva-acidified HMOs and found that acidic HMOs promoted the production of IFN-γ and IL-10, inhibited the Th2 response, and shifted the immune response to a more balanced Th1/Th2 paradigm, which resulted in enhanced defenses against pathogenic infections and attenuated the neonatal intestinal mucosa’s susceptibility to food allergies and autoimmune diseases.^132^

In addition, *B. longum* uses the metabolites produced by HMOs to regulate the immune system indirectly. For example, lactate allows CD4+ T cells to convert to IL-17+ T cell subsets and reduces CD8+ T cell cytolytic capacity, regulating the immune response. Propionate interacts with GPR43, mediating the AMP-activated protein kinase (AMPK) signaling pathway to increase AKT (phosphokinase B) phosphorylation, thereby modulating mucosal immunity. Butyrate also inhibits the recruitment and proinflammatory activity of neutrophils, macrophages, dendritic cells, and effector T cells.^133^ Finally, it is implicated in increasing the number and activity of regulatory T cells. ILA derived from *B. longum* subsp. *infantis* EVC001 upregulated immune regulatory galectin-1 in Th 2 and Th 17 cells during polarization and galectin-1 further bound HMOs.^102^

## 7. Potential application and research limitation of HMOs combined with *B. longum* as synbiotics

**Figure 5.**
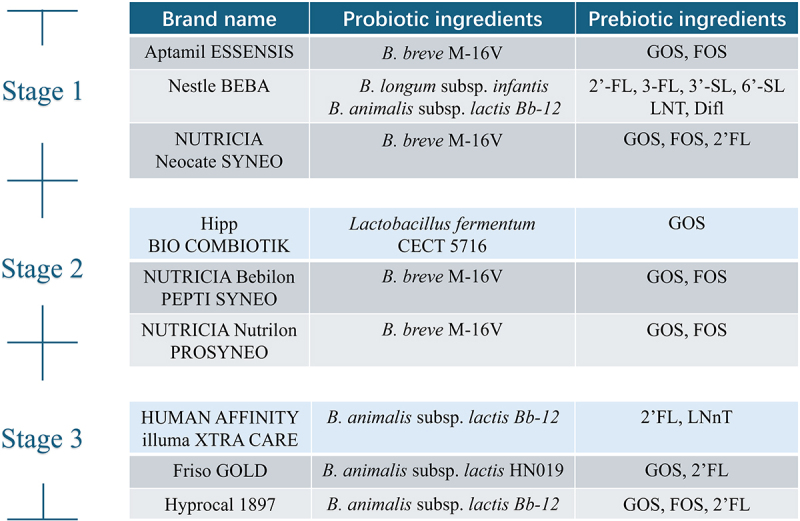
Market infant formula (stage 1-3) supplemented with probiotics and prebiotics simultaneously. LNT: lacto-N-tetraose; 2’-FL: 2’-fucosyllactose; 3-FL: 3-fucosyllactose; 3’-SL: 3’-sialyllactose; 6’-SL: 6’-sialyllactose; Difl: Difucosyllactose; GOS: Galactooligosaccharides; FOS: Fructooligosaccharides.

Synbiotics, a combination of probiotics and prebiotics, act synergistically to provide enhanced health benefits over either prebiotic or probiotic taken separately.^134^ The metabolites produced by probiotics after consumption of prebiotics are small in size and are thought to diffuse through intestinal cells into the bloodstream, positively affecting health regulation throughout the body.^135^ The World Health Organization (WHO) and the United Nations Children’s Fund (UNICEF) recommend exclusive breastfeeding for the first 6 months of life, followed by the introduction of complementary foods after 6 months, and continued breastfeeding until 2 years of age or beyond.^136^ Consequently, we have counted the market formula brands of one to three-stage (0–36 months) containing synbiotics (Figure 5).

Prebiotic ingredients commonly used in infant formulas include GOS and fructooligosaccharides (FOS), and the HMOs fortified focused mainly on 2‘-FL, as well as probiotics such as *Bifidobacterium* and *Lactobacillus*. The substitution of GOS and FOS for HMOs is controversial. Some studies suggest that HMOs are irreplaceable and that there is a significant difference in metabolites produced by infants fed human milk compared to infants fed GOS-containing formulas, differences that can be reduced by supplementation with HMOs.^137^ However, other studies have shown that GOS and 2‘-FL have complementary-release sites in the colon and that supplementation with both in infant formulas has better health benefits. Therefore, in the future, it will be necessary to consider whether infant formulas should be supplemented only with HMOs or with both HMOs, GOS and FOS in a certain ratio, considering health benefits and economics. Additionally, we observed that the main strains currently added to infant formulas focus on *B.* *animalis* subsp. *lactis* and *B. breve*. However, their utilization of HMOs is more limited compared to *B. longum* (especially *B. longum* subsp. *infantis*).^138^ Thus, we believe that one of the ways to improve the utilization of HMOs in infants could be achieved by supplementing HMOs with *B. longum* as synbiotics. Furthermore, since most infant formulas are also supplemented with HMOs, GOS and FOS simultaneously and the main source of FOS is from plants, the combination of *B. longum* subsp. *longum* (high utilization ability for plant polysaccharides) and *B. longum* subsp. *infantis* could further increase the utilization rate of prebiotics in the infant formula and produce more metabolites beneficial to the infants’ intestinal tract.

However, limitations are still present in current studies on *B. longum* and HMOs as synbiotics. First, based on previous research, we found that the process of how *B. longum* metabolizes different HMOs and the specific mechanism of action of the metabolites in the infants’ gut are poorly understood due to the limited number of synergistic studies. We believe that the composition of HMOs is complex and variable, and thus, the effects of different types of HMOs on the colonization of different bifidobacteria and their interactions need to be further explored in future studies. Next, we know that the contents of HMOs in breast milk show dynamic changes, but the HMOs added to current infant formulas are relatively homogeneous in composition and fixed in contents. This raises the question of whether different concentrations of HMOs should be added to infant formulas at different stages to better meet infants’ nutritional needs. Finally, it has been hypothesized that the presence of too many HMOs consumer-*Bifidobacterium*, which may be detrimental to health because of disrupting the immune regulation of HMOs.^103^ Therefore, the ratio of bifidobacterial cells to HMOs needs further clarification to provide a theoretical basis for the future use of them as symbiotic in functional foods.

## 8. Conclusion

*Bifidobacterium* has a versatile ability to utilize carbohydrates, and as a recognized excellent “pioneer colonizer” in the infant gut, it has been shown to play a positive role in intestinal disorders, immunity, and even neurological development. As a natural prebiotic with a diverse and irreplaceable structure, HMOs can be used as a bifidogenic factor to stimulate the growth of *B. longum*, one of the main colonizing species of the early intestinal tract. Synergizing HMOs with *B. longum* may increase beneficial metabolites to protect the intestinal barrier and promote the maturation of the infant’s immune system, which in turn has the potential to prevent NEC, infections, and food allergies. The addition of HMOs and *B. longum* as synbiotics to infant formulas, particularly in cases of inadequate breast milk supply, may provide opportunities to improve infant health and have promising application prospects.
